# Complete genome sequencing and analysis of six enterovirus 71 strains with different clinical phenotypes

**DOI:** 10.1186/1743-422X-10-115

**Published:** 2013-04-11

**Authors:** Hong-ling Wen, Lu-ying Si, Xiao-jing Yuan, Shu-bin Hao, Feng Gao, Fu-lu Chu, Cheng-xi Sun, Zhi-yu Wang

**Affiliations:** 1 The Key Laboratory of experimental teratology of Ministry of Education, Department of Virology, School of Public Health, Shandong University, Jinan, Shandong, 250012, People’s Republic of China; 2 Shandong Medical Equipment Quality Supervision And Inspection Center, Jinan, Shandong, 250012, People’s Republic of China; 3 Linyi People's Hospital, Linyi, Shandong, 276000, People’s Republic of China

**Keywords:** Enterovirus 71, Virulent determinant, Hand, foot and mouth disease

## Abstract

**Background:**

Hand, foot and mouth diseases (HFMD) caused by enterovirus 71(EV71) presents a broad spectrum of clinical manifestations ranging from mild febrile disease to fatal neurolocal disease. However, the mechanism of virulence is unknown.

**Methods:**

We isolated 6 strains of EV71 from HFMD patients with or without neurological symptoms, and sequenced the whole genomes of the viruses to reveal the virulence factors of EV71.

**Results:**

Phylogenetic tree based on VP1 region showed that all six strains clustered into C4a of C4 sub-genotype. In the complete polypeptide, 298 positions were found to be variable in all strains, and three of these positions (Val^P814^/Ile^P814^ in VP1, Val^P1148^/Ile^P1148^ in 3A and Ala ^P1728^/Cys ^P1728^/Val ^P1728^ in 3C) were conserved among the strains with neurovirulence, but variable in strains without neurovirulence. In the 5^′^-UTR region, it showed that the first 10 nucleotides were mostly conserved, however from the 11th nucleotide, nucleotide insertions and deletions were quite common. The secondary structure prediction of 5^′^-UTR sequences showed that two of three strains without neurovirulence (SDLY11 and SDLY48) were almost the same, and all strains with neurovirulence (SDLY96, SDLY107 and SDLY153) were different from each other. SDLY107 (a fatal strain) was found different from other strains on four positions (C^P241^/T^P241^, A^P571^/T^P571^, C^P579^/T^P579^ in 5^′^-UTR and T^P7335^/C^P7335^ in 3^′^-UTR).

**Conclusions:**

The three positions (Val^P814^/Ile^P814^ in VP1, Val^P1148^/Ile^P1148^ in 3A and Ala ^P1728^/Cys ^P1728^/Val ^P1728^ in 3C), were different between two phenotypes. These suggested that the three positions might be potential virulent positions. And the three varied positions were also found to be conserved in strains with neurovirulence, and variable in strains without neurovirulence. These might reveal that the conservation of two of the three positions or the three together were specific for the strains with neurovirulence. Varation of secondary structure of 5^′^-UTR, might be correlated to the changes of viral virulence. SDLY107 (a fatal strain) was found different from other strains on four positions, these positions might be related with death.

## Background

Enterovirus 71 (EV71) belongs to the Enterovirus genus of the family Picornaviridae. It is one of the pathogens that are associated with hand, foot and mouth disease (HFMD). In most cases, EV71 infections are generally mild. However, this virus has also been implicated to cause severe neurological manifestations including aseptic meningitis, polio-like paresis and possibly fatal encephalitis [1].

Since 1969, when EV71 was first isolated in California, USA [2], EV71 associated outbreaks have been reported worldwide [3-10]. In recent years, it has gained more attention as there is an upward trend in the prevalence of EV71 in Asia [11]. EV71 infection is a serious threat to the health of infants and young children; therefore, it is necessary to understand the mechanism of central nervous system involvement. Zheng et al. reported nucleotide differences in 5^′^-UTR between strains isolated from patients with and without neurological symptom, and proposed that such variation may be correlated with different clinical presentations [12]. Shih-Cheng Chang reported that a significant amino acid change was observed in more than one of high virulent strains [13]. Melchers et al. suggested that point mutations in 3^′^-UTR can result in a lethal phenotype [14]. All these points were located in different regions of the genome, therefore, it is necessary to search for potential points associated with neurovirulence in complete genome.

## Results

### Virus identification and segmented amplification

**Figure 1:**
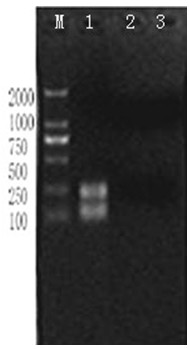
**Amplification of nucleic acid of SDLY107 by RT-PCR.** M: DL2000 Marker, Lane 1: SDLY107, Lane 2: cell control, Lane 3: reagent control.

**Figure 2:**
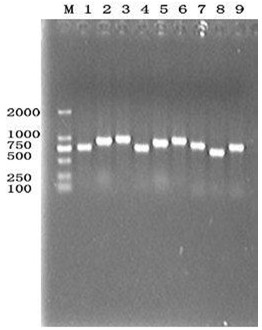
**Segmented amplification of complete sequence of SDLY107 strain by RT-PCR.** M: DL2000 Marker, Lane 1:1 ~ 769 bp, Lane 2:719 ~ 1632 bp, Lane 3:1597 ~ 2568 bp, Lane 4:2532 ~ 3346 bp, Lane 5:3216 ~ 4134 bp, Lane 6:4043 ~ 5045 bp, Lane 7:4969 ~ 5877 bp, Lane 8:5799 ~ 6567 bp, Lane 9:6500 ~ 7405 bp.

All the six strains were proved to be EV71 by RT-PCR (Figure 1), and were amplified with the nine pairs of overlapping primes (Figure 2).

### Sequence analysis of the genomes

The sequences of the six strains were desposited in GenBank (GenBank accession number JX244182, JX244183, JX244184, JX244185, JX244186, JX244187). The genomes of strains SDLY11, SDLY48, SDLY96 and SDLY107 were all 7405 bp in length, whereas strains SDLY1 and SDLY153 were 7408 bp in length. All six strains had one ORF which encoded a polypeptide of 2193 amino acids.

Pair-wise nucleotide and amino acid sequence comparisons showed that the genetic variation among the six strains was limited. The nucleotide homology of the genomes was 95.5% ~ 99.7%. The amino acid homology of the polyproteins was 98.5% ~ 99.5%. The nucleotide homology of 5^′^-UTR and 3^′^-UTR were 97.2% ~ 99.6% and 95.3 ~ 100.0%, respectively. They shared 77.5%-99.0% nucleotide homology of the genomes with reference strains, and 98.6% to 89.6% at the amino acid level (Table 1).

**Table 1:** The nucleotide and amino acid homology of the six strains with reference strains(nucleotide/amino acid)

	**SDLY1**	**SDLY11**	**SDLY48**	**SDLY96**	**SDLY107**	**SDLY153**
U22521(A)	80.1/95.1	79.9/95.1	79.9/95.2	79.9/95.0	79.9/95.0	79.9/94.5
ETU22522(B)	81.9/95.9	81.9/95.8	81.9/95.9	81.9/95.8	82.0/95.7	81.7/95.2
DQ341361(C1)	82.3/96.3	82.2/96.2	82.5/96.4	82.3/96.2	82.3/96.2	82.1/95.6
AF119795(C2)	81.8/93.0	81.4/93.0	81.9/93.0	81.6/93.0	81.3/93.0	81.3/92.6
DQ341356(C3)	81.8/96.2	81.4/96.2	81.9/96.2	81.6/96.2	81.3/96.2	81.3/95.6
EU703813(C4a)	97.2/99.2	98.9/99.6	96.4/99.1	99.0/99.6	98.6/99.7	98.4/99.1
AF302996(C4b)	91.3/95.9	91.0/95.9	91.4/95.8	91.0/95.9	90.8/95.8	90.8/95.2
EU527983(C5)	82.1/96.4	82.0/96.4	82.3/96.3	82.1/96.4	82.1/96.5	82.1/95.9
U05876(CA16)	77.9/90.3	77.6/90.2	77.7/90.2	77.7/90.2	77.9/90.2	77.5/89.6

**Figure 3:**
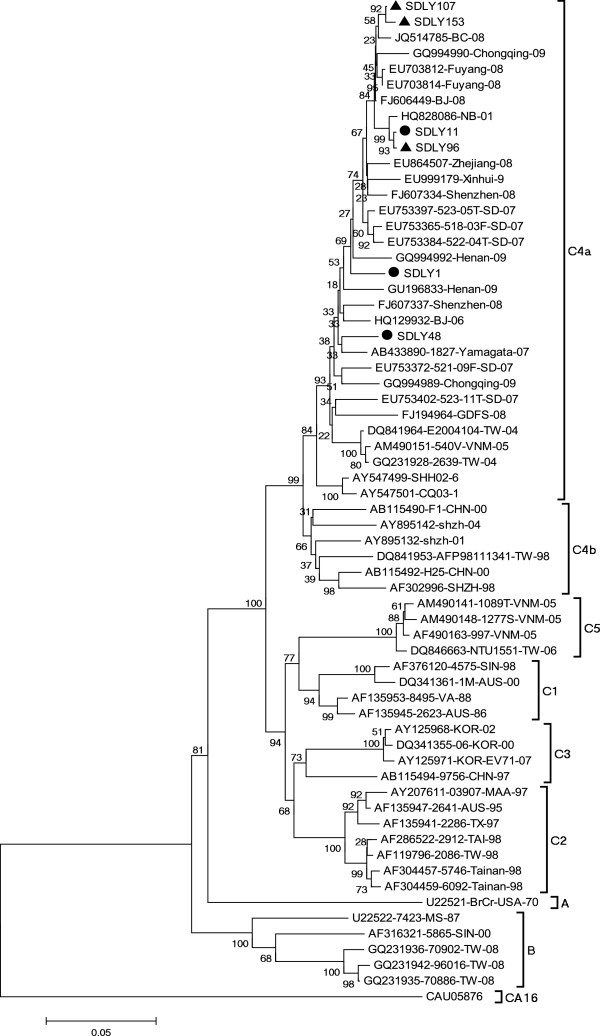
**Phylogenetic tree hylogenetic analysis based on EV71 VP1 nucleotide sequences (891 bp).** ● strains isolated from patients without neurovirulence in this study, ▲ strains isolated from patients with neurological symptom in this study. The phylogenetic tree was drawn using the neighbor joining method. Bootstrap values are shown as percentages derived from 1000 samplings and the scale reflects the number of nucleotide substitution per site along the branches.

Phylogenetic analysis of the six strains and reference strains based on the nucleotide sequences of the complete VP1 region showed that all the six strains clustered in the C4a of C4 sub-genotype (Figure 3).

### Analysis of polyprotein

The polyprotein consists in three regions [15]: P1 containing capsid proteins (VP ~ VP4), P2 and P3 containing non-structural proteins (2A, 2B, 2C, 3A, 3B, 3C and 3D) which are crucial for virus replication.

The complete genome sequence of 58 strains of EV71 were available in GenBank, but only 25 strains had information of clinical symptoms of the patients. 13 of the 25 strains isolated from patients with neurological symptom, and 12 of the strains were isolated from patients without neurological symptom. As we aimed to correlated sequences of defined clinical symptoms, we only analyzed the 25 genomes of EV71 strains from GenBank that had description of clinical symptoms and the 6 strains sequenced in this study (Table 2). In the complete polyprotein, 298 positions were found to be variable and three of these positions were statistical significant (Table 3). The three points were Val^P814^/Ile^P814^ (Fisher^′^s Exact Test, *P* = 0.018, a =0.05), Val^P1148^/Ile^P1148^ (Fisher^′^s Exact Test, *P* = 0.043, a =0.05) and Ala ^P1728^/Cys ^P1728^/Val ^P1728^ (Fisher^′^s Exact Test, *P* = 0.018, a =0.05).

**Table 2:** Complete genome sequences of 31 strain used in this study

**Strains with neurovirulence**		**Strains without neurovirulence**	
**GenBank No.**	**Source**	**GenBank No.**	**Source**
U22522	GenBank	EU753384	GenBank
AF316321	GenBank	HQ129932	GenBank
JQ514785	GenBank	AF304459	GenBank
EU753365	GenBank	GQ994989	GenBank
GQ231942	GenBank	FJ607334	GenBank
HQ828086	GenBank	GQ231936	GenBank
GU196833	GenBank	AF119796	GenBank
EU753397	GenBank	AF304457	GenBank
GQ994992	GenBank	GQ231935	GenBank
GQ231928	GenBank	GQ994990	GenBank
EU703814	GenBank	FJ606449	GenBank
FJ607337	GenBank	DQ341361	GenBank
EU703812	GenBank	SDLY1	Isolated in this study
SDLY96	Isolated in this study	SDLY11	Isolated in this study
SDLY107	Isolated in this study	SDLY48	Isolated in this study
SDLY153	Isolated in this study		

**Table 3:** Significant positions of polyprotein of 31 strains

**Region**	**Position**	**Amino acid**	
		**Strains with neurovirulence**	**Strains without neurovirulence**
VP1	814	Val/Ile(16/0)*	Val/Ile(10/5)
3A	1148	Val/Ile(16/0)	Val/Ile(11/4)
3C	1728	Ala/Cys/Val (16/0/0)	Ala/Cys/Val (10/1/4)

*Val/Ile(16/0): in all 16 strains with neurovirulence, 16 of them were Val^P814^ and none of them was Ile^P814^

### Analysis of 5^′^-UTR

5^′^-UTR sequences of 31 strains were aligned. It showed that the first 10 nucleotides were mostly conserved, however from the 11th nucleotide, nucleotide insertions and deletions were quite common. No position was found statistical significantly different between strains with and without neurological symptom. Whereas SDLY107 (a fatal strain) was found different from other strains on three positions (C^P241^/T^P241^, A^P571^/T^P571^, C^P579^/T^P579^), suggesting that these positions might be related to death.

**Figure 4:**
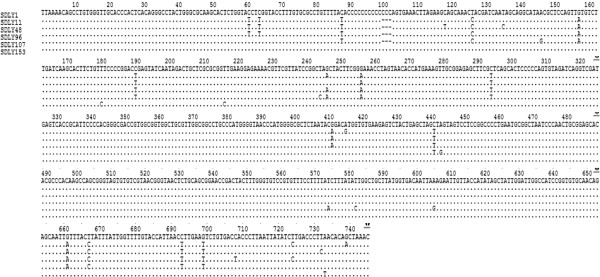
**Sequence alignment of 5**^**′**^**-UTR sequences of 6 strains isolated in our study.**

5^′^-UTR sequences of 6 strains isolated in our study were aligned by BioEdit 7.09 software (Figure 4), and no significantly difference was found.

**Figure 5:**
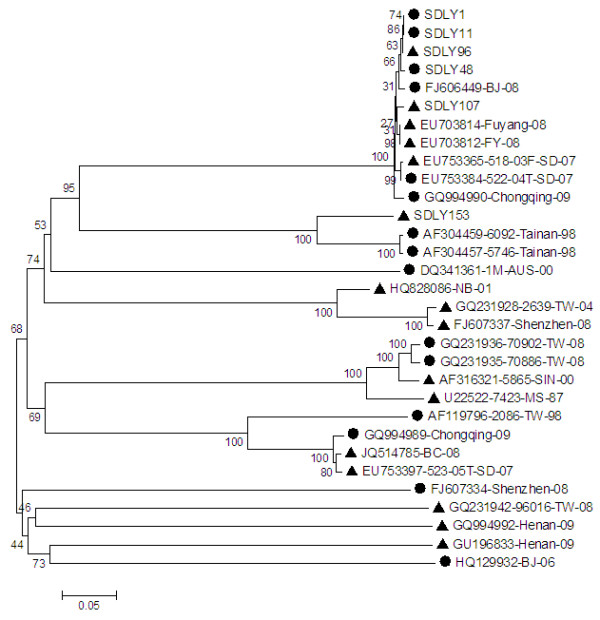
**Phylogenetic tree hylogenetic analysis based on 5**^**′**^**-UTR nucleotide sequences.** ● strains without neurovirulence, ▲ strains with neurological symptom. The phylogenetic tree was drawn using the neighbor joining method. Bootstrap values are shown as percentages derived from 1000 samplings and the scale reflects the number of nucleotide substitution per site along the branches.

Phylogenetic analysis of 31 strains based on the nucleotide sequences of 5^′^-UTR showed no definite regularity of these strains, revealing that there was no distinction on evolution between strains with different symptoms (Figure 5).

**Figure 6:**
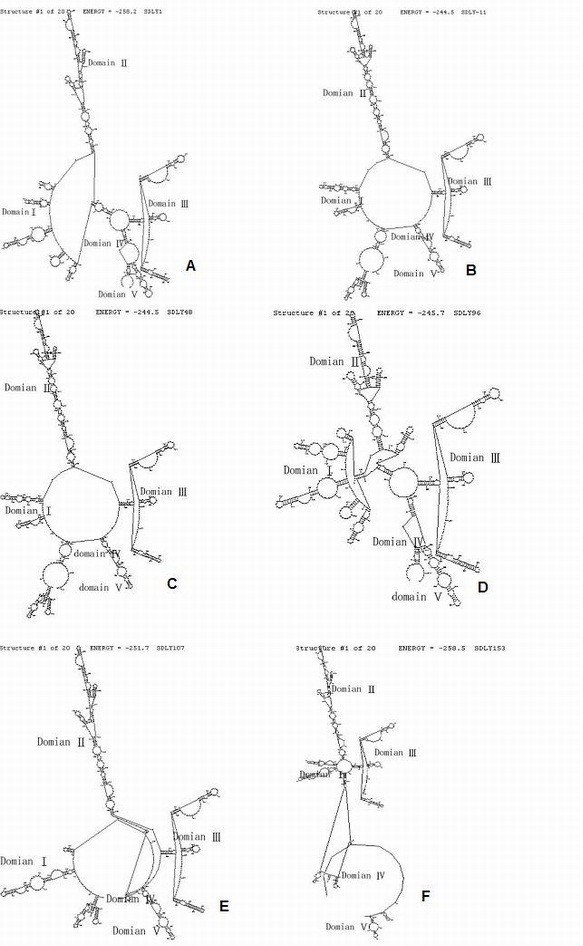
**The secondary structures of 5**^**′**^**-UTR. A**: SDLY1, **B**: SDLY11, **C**: SDLY48, **D**: SDLY96, **E**: SDLY107, **F**: SDLY153.

The 5^′^-UTR of EV71 could be divided into two regions: the 5^′^ terminal cloverleaf and the IRES element [16]. IRES initiated genome translation by a cap-independent mechanism mediated [17]. IRES includes five stem loop (domain I ~ V), all of these five domains are essential to viral RNA replication and translation. The secondary structure prediction of the complete 5^′^-UTR sequences showed that two of three strains from patents without neurological symptom (SDLY11 and SDLY48) were almost the same, and all strains with neurovirulence (SDLY96, SDLY107 and SDLY153) were different from each other (Figure 6). In IRES element, domain III and II were relatively conservative regions, however, domainI, IV and V were marked variation. This information suggested that variety of secondary structure of 5^′^-UTR, especially domainI,IV and V might influence virulence.

### Analysis of 3^′^-UTR

The 3^′^-UTR of EV71 was a highly conserved region and point mutations in the 3^′^-UTR could result in a lethal phenotype [14]. Alignment of 3^′^-UTR sequences of 31 strains by BioEdit 7.09 software did not reveal significant position associated with virulence. However, SDLY107 (a fatal strain) was found different from other strains on position T^P7335^/C^P7335^, suggesting that this position might be correlated to death.

**Figure 7:**
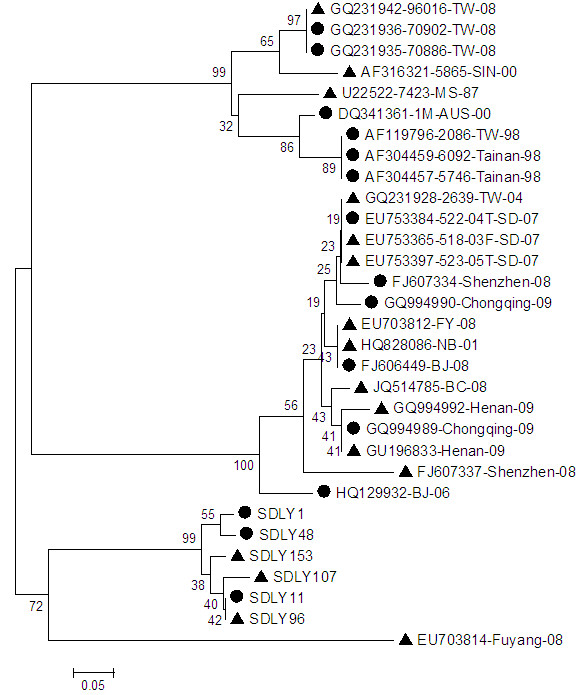
**Phylogenetic tree hylogenetic analysis based on 3**^**′**^**-UTR nucleotide sequences.** ● strains without neurovirulence, ▲ strains with neurological symptom. The phylogenetic tree was drawn using the neighbor joining method. Bootstrap values are shown as percentages derived from 1000 samplings and the scale reflects the number of nucleotide substitution per site along the branches.

Phylogenetic analysis of 31 strains based on the nucleotide sequences of 3^′^-UTR (Figure 7) showed strains with different symptoms were mixed up, suggesting that there was no distinction on evolution between strains with or without neurological symptoms.

**Figure 8:**
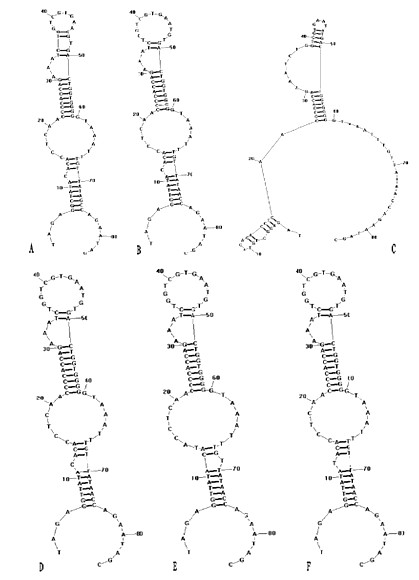
**The secondary structures of 3**^**′**^**-UTR. A**: SDLY1, **B**: SDLY11, **C**: SDLY48, **D**: SDLY96, **E**: SDLY107, **F**: SDLY153.

The secondary structure prediction of the complete 3^′^-UTR sequences showed that except strain SDLY48, the other five strains were almost the same (Figure 8).

## Discussion

EV71 is one of the most virulent enteroviruses and can cause mortality in children [1]. Defining virulent positions on molecular level is considered as one of the most important aspects of disease prevention. In our study, complete genomes of six EV71 strains with different clinical phenotypes were sequenced and analyzed. Together with other strains isolated in Shandong in recent years, the six strains clustered into C4a of C4 sub-genotype [18].

At present, molecular neurovirulence determinant of EV71 remains unclear, though virulence factors of other enteroviruses have been reported. Nucleotide 480, 481 and 472 on 5^′^-UTR of poliovirus were identified as neurovirulence determinants of poliovirus [19-21]. Minetaro et al. reported that mutation of the EV71 standard strain BrCr in 5^′^-UTR showed attenuated neurovirulence in the cynomolgus monkey model [22]. In this study, insertions and deletions were frequently found in 5^′^-UTR region. Two of three EV71 strains (SDLY11 and SDLY48) from patients without neurovirulence had almost the same secondary structure of 5^′^-UTR, and all strains with neurovirulence (SDLY96, SDLY107 and SDLY153) were different from each another. In IRES element, domain III and II were relatively conserved regions, however, domainI, IV and V are very variable. These suggest that variation of the secondary structure of the 5^′^-UTR, especially domainI, IV and V might be correlated to the virulence. When aligned the strain isolated from a fatal patient (SDLY107) with other five strains, three position of 5^′^-UTR (C^P241^/T^P241^, A^P571^/T^P571^, C^P579^/T^P579^) might be related to the virulence.Li et al. reported that four amino acids (Gly^P710^/Gln^P710^/Arg^P710^ and Glu^P729^) in the DE and EF loop of VP1, one (Lys^P930^) in the surface of protease 2A were potentially associated with EV71 virulence [23]. In our study, three positions, Val^P814^/Ile^P814^ in VP1, Val^P1148^/Ile^P1148^ in 3A and Ala ^P1728^/Cys ^P1728^/Val ^P1728^ in 3C, were different between two phenotypes. These results suggest that three positions are potential virulent positions. The position 814 locates in C-terminal part of the VP1 protein which locates on the surface of the virus, mediates the initiation of infection by binding to receptors on the host membrane [24]. C-terminal part of the VP1 protein were supposed to be capable of eliciting neutralizing antibodies against EV71 [25]. Variations in VP1 region may influence the ability of the virus binding to host cell and eliciting neutralizing antibodies. Protein 3A plays a role in inhibiting cellular protein secretion and mediating presentation of membrane proteins during viral infection. Variations in 3A region may affect the process of viral infection. Protein 3C can cleave numerous factors and regulators that are associated with cellular DNA-dependant RNA polymerase I, II and III, and may be involved in the virus-induced blockage of host transcription. Variations in 3C region may affect activity of RNA polymerase and host cellular transcription. The three positions were conserved in strains with neurovirulence, and variable in strains without neurovirulence. These also reveales that the conservation of two of the three positions or the three together maybe specific for the strains with neurovirulence.

The 3^′^-UTR is a highly conserved domain and mutations in the 3^′^-UTR may cause change of phenotype. However, in our study, analysis of nucleotides of 3^′^-UTR showed no virulence associated nucleotides.

To test our aforementioned findings, site-directed mutagenesis need to be performed on these positions in the future study, and infectious cDNA clones with different potential virulent positions need to be constructed and evaluated at *ex vivo* and *in vitro*.

## Methods

### Cells and viruses

EV71 strains SDLY1, SDLY11, SDLY48, and SDLY96 were isolated from stool samples of four patients without neurovirulence. SDLY107, SDLY153 were isolated from anal swabs samples of two patients. Among these strains, SDLY1, SDLY11 and SDLY48 were isolated from patients with mild symptoms. SDLY96 and SDLY153 were isolated from patients with neurological symptom, and SDLY107 was isolated from a fatal patient. All six patients were from Linyi City, Shandong Province, China. Human rhabdomyosarcoma (RD) cells were maintained in DMEM supplemented with 10% FBS. Viruses were propagated on RD cells to increase the titer for use in subsequent assays.

### RNA extraction and virus identification

Total virus RNAs were extracted from EV71-infected cell culture supernatants using a RNA extraction kit (OMEGA) following the manufacture^′^s instructions. Virus types were identified by One-Step RT-PCR described previously [26].

### Segmented amplification of the complete genomes

Nine overlapping clones covering the whole viral genome were obtained by RT-PCR (QIAGEN, OneStep RT-PCR Kit). RT-PCR amplifications were carried out with the primers in Table 4. RT-PCR products were purified using Gel Extraction Mini Kit (OMEGA) and were cloned to the pMD19-T plasmid (TaKaRa). The recombinant vectors were transformed into competent *E*. coli DH5α. Positive clones were sequenced by Biosune Biotechnology Co. Ltd.

**Table 4:** Primers used for amplifying the genome

**NO.**	**Name of primer**	**Sequence**	**Amplification(bp)**
1	P1+	TTAAAACAGCCTGTGGGTTGCACC	769 bp
	P769-	GTGTAGACACTTGCGAACC	
2	P719+	CTTGACCCTTAACACAGCTA	914 bp
	P1632-	GCTCCTTGGTCGTAGTCTAG	
3	P1597+	GTGCCTATTAGCCCACTAGAC	973 bp
	P2568-	ACCTTGCCTGTATCCAGTCGA	
4	P2532+	ACAGGTGAGCAGTCATCGACT	815 bp
	P3346-	CTGTTGTCCAAATTTCCCAAGA	
5	P3216+	GTGGATACCTCGCCCGATGC	919 bp
	P4134-	CACTCTAACCCCTTAGCGGCGT	
6	P4043+	CCATCTTAGGTATCCCTATCGC	1003 bp
	P5045-	TTGTGTTGCCAATGGCGGACC	
7	P4969+	GTCAGATACAGTGTGGATACG	909 bp
	P5877-	CCACCAATGTGAATACCGACAA	
8	P5799+	CAACTTTCCTACTAAAGCAGGAC	769 bp
	P6567-	CCCACGGCTGATCCAGTTATCG	
9	P6500+	TGGCTTTCGGACATTTGTATGA	906 bp
	P7405-	GCTATTCTGGTTATAACAAATTTACC	

### Sequences analysis

The nucleotide sequences of six complete genomes and the derived amino acid sequences were analyzed by BioEdit 7.09 software. The genotype and subgenotype were determined by comparing sequences with reference strains from GenBank. The secondary structures of 5^′^-UTR and 3^′^-UTR were predicted by RNA structure 4.0 software. The phylogenetic tree was constructed using MEGA 4 software based on the nucleotide sequences of the complete VP1 region.

### Ethics statement

This study was approved by the ethical committees of School of Public Health, Shandong University, Jinan, Shandong 250012, China (permit number 20080301). Written consents were obtained from all children^′^s parents involved in the study.

## Abbreviations

EV71: Enterovirus 71; HFMD: Hand, foot and mouth diseases; RT-PCR: Reverse transcription-polymerase chain reaction; ORF: Open reading frame; IRES: Internal ribosome entry site; DMEM: Dulbecco’s modified Eagle’s medium; FBS: Supplemented with 10% fetal bovine serum.

## Competing interests

The authors declare that they have no competing interests.

## Authors’ contributions

HLW, ZYW and SBH conceived the study and designed the experiments. LYS, XJY, FLC, CXS performed the experiments. HLW and LYS analyzed the data and wrote the manuscript. FG contributed in sample collection. All authors read and approved the final manuscript.

## References

[B1] Hagiwara A Yoneyama T Takami S Hashimoto I Genetic and phenotypic characteristics of enterovirus 71 isolates from patients with encephalitis and with hand, foot and mouth disease Arch Virol 1984 79 3–4 273 283 6320781 10.1007/BF01310816

[B2] Blomberg J Lycke E Ahlfors K Johnsson T Wolontis S von Zeipel G New enterovirus type associated with epidemic of aseptic meningitis and or hand, foot, and mouth disease Lancet 1974 2 7872 112 4136956 10.1016/s0140-6736(74)91684-5

[B3] Chumakov M Voroshilova M Shindarov L Shindarov L Lavrova I Enterovirus 71 isolated from cases of epidemic poliomyelitis-like disease in Bulgaria Arch Virol 1979 60 3–4 329 340 228639 10.1007/BF01317504

[B4] Ishimaru Y Nakano S Yamaoka K Takami S Outbreaks of hand, foot, and mouth disease by enterovirus 71. High incidence of complication disorders of central nervous system Arch Dis Child 1980 55 8 583 588 10.1136/adc.55.8.583 6254449 PMC1627055

[B5] Lan YC Lin TH Tsai JD Yang YC Peng CT Molecular epidemiology of the 2005 enterovirus 71 outbreak in central Taiwan Scand J Infect Dis 2011 43 5 345 349 10.3109/00365548.2010.545995 21231813

[B6] Kim KH Enterovirus 71 infection: An experience in Korea 2009 Korean J Pediatr 2010 53 5 616 622 10.3345/kjp.2010.53.5.616 21189926 PMC2994121

[B7] Li Wei A Benjamin KW K Kwai Peng C Chua LT James L Goh KT Epidemiology and Control of Hand,Foot and Mouth Disease in Singapore, 2001–2007 Ann Acad Med Singapore 2009 38 2 106 112 19271036

[B8] Schuffenecker I Mirand A Antona D Henquell C Chomel JJ Epidemiology of human enterovirus 71 infections in France, 2000–2009 J Clin Virol 2011 50 1 50 56 10.1016/j.jcv.2010.09.019 21035387

[B9] McMinn P Stratov I Nagarajan L Davis S Neurological manifestations of enterovirus 71 infection in children during an outbreak of hand, foot, and mouth disease in Western Australia Clin Infect Dis 2001 32 2 236 242 10.1086/318454 11170913

[B10] Chan LG Parashar UD Lye MS Ong FG Zaki SR Deaths of children during an outbreak of hand, foot, and mouth disease in sarawak, malaysia: clinical and pathological characteristics of the disease. For the Outbreak Study Group Clin Infect Dis 2000 31 3 678 683 10.1086/314032 11017815

[B11] Komatsu H Shimizu Y Takeuchi Y Ishiko H Takada H Outbreak of severe neurologic involvement associated with enterovirus 71 infection Pediatr Neuro 1999 20 1 17 23 10.1016/S0887-8994(98)00087-3 10029254

[B12] Zheng ZM He PJ Caueffield D Neumann M Specter S Enterovirus 71 isolated from China is serologically similar to the prototype E71 BrCr strain but differs in the 5′-noncoding region J Med Virol 1995 47 2 161 167 10.1002/jmv.1890470209 8830120

[B13] Chang SC Li WC Chen GW Tsao KC Huang CG Genetic characterization of enterovirus 71 isolated from patients with severe disease by comparative analysis of complete genomes J Med Virol 2012 84 6 931 939 10.1002/jmv.23287 22499017

[B14] Melchers WJ Hoenderop JG Bruins Slot HJ Pleij CW Pilipenko EV Kissing of the two predominant hairpin loops in the coxsackie B virus 3′untranslated region is the essential structural feature of the origin of replication required for negative-strand RNA synthesis J Virol 1997 71 1 686 696 8985400 10.1128/jvi.71.1.686-696.1997 PMC191101

[B15] Wimmer E Hellen CU Cao X Genetics of poliovirus Annu Rev Genet 1993 27 353 436 10.1146/annurev.ge.27.120193.002033 8122908

[B16] Bedard KM Semler BL Regulation of picornavirus gene expression Microbes Infect 2004 6 7 702 713 10.1016/j.micinf.2004.03.001 15158778

[B17] Pelletier J Kaplan G Racaniello VR Sonenberg N Cap-independent translation of poliovirus mRNA is conferred by sequence elements within the 5′noncoding region Mol Cell Biol 1988 8 3 1103 1112 2835660 10.1128/mcb.8.3.1103-1112.1988 PMC363253

[B18] Liu XL Wang ZG Yang TT Yi T Molecular epidemiology of human enterovirus 71 strains in Qingdao region, Shandong province, 2007–2009 Chin J Epidemiol 2011 32 4 382 384 21569671

[B19] Evans DM Dunn G Minor PD Schild GC Cann AJ Increased neurovirulence associated with a single nucleotide change in a noncoding region of the Sabin type 3 poliovaccine genome[J] Nature 1985 314 6011 548 550 10.1038/314548a0 2986004

[B20] Guillot S Otelea D Delpeyroux F Crainic R Point mutations involved in the attenuation/neurovirulence alternation in type 1 and 2 oral polio vaccine strains detected by site-specific polymerase chain reaction Vaccine 1994 12 6 503 507 10.1016/0264-410X(94)90307-7 8036823

[B21] Rezapkin GV Fan L Asher DM Fibi MR Dragunsky EM Mutations in Sabin 2 strain of poliovirus and stability of attenuation phenotype Virology 1999 258 1 152 160 10.1006/viro.1999.9718 10329577

[B22] Arita M Shimizu H Nagata N Ami Y Suzaki Y Temperature-sensitive mutants of enterovirus 71 show attenuation in cynomolgus monkeys J Gen Virol 2005 86 5 1391 1401 10.1099/vir.0.80784-0 15831951

[B23] Li R Zou Q Chen L Zhang H Wang Y Molecular Analysis of Virulent Determinants of Enterovirus 71 PLoS One 2011 6 10 e26237 10.1371/journal.pone.0026237 22039449 PMC3198388

[B24] Lin JY Chen TC Weng KF Chang SC Chen LL Viral and host proteins involved in picornavirus life cycle J Biomed Sci 2009 16 1 103 10.1186/1423-0127-16-103 19925687 PMC2785775

[B25] Foo DG Alonso S Phoon MC Ramachandran NP Chow VT Identification of neutralizing linear epitopes from the VP1 capsid protein of Enterovirus 71 using synthetic peptides Virus Res 2007 125 9 61 68 17222936 10.1016/j.virusres.2006.12.005

[B26] Guidelines for the prevention and treatment of HFMDhttp://www.moh.gov.cn/publicfiles/business/cmsresources/wsb/cmsrsdocument/doc4510.doc

